# New Insights into HIV/AIDS-Associated Cryptococcosis

**DOI:** 10.1155/2013/471363

**Published:** 2013-02-25

**Authors:** Spinello Antinori

**Affiliations:** Department of Biomedical and Clinical Sciences Lvigi Sacco, Università degli Studi di Milano, Via GB Grassi 74, 20157 Milano, Italy

## Abstract

Cryptococcal meningitis is a life-threatening opportunistic fungal infection in both HIV-infected and HIV-uninfected patients. According to the most recent taxonomy, the responsible fungus is classified into a complex that contains two species (*Cryptococcus neoformans* and *C. gattii*), with eight major molecular types. HIV infection is recognized worldwide as the main underlying disease responsible for the development of cryptococcal meningitis (accounting for 80–90% of cases). In several areas of sub-Saharan Africa with the highest HIV prevalence despite the recent expansion of antiretroviral (ARV) therapy programme, cryptococcal meningitis is the leading cause of community-acquired meningitis with a high mortality burden. Although cryptococcal meningitis should be considered a neglected disease, a large body of knowledge has been developed by several studies performed in recent years. This paper will focus especially on new clinical aspects such as immune reconstitution inflammatory syndrome, advances on management, and strategies for the prevention of clinical disease.

## 1. Introduction

The encapsulated yeast, *Cryptococcus* spp., is a major cause of fungal meningitis and meningoencephalitis especially in immunocompromised patients [1, 2]. This basidiomycete fungus was first isolated in 1894 by Sanfelice in fruit juices and subsequently recovered from the tibial lesion of a patient by Busse and Buschke [3–5]. The genus has been named from the Greek words, *kryptos* (hidden) and *kokkos* (berry) but a long lists of names and synonyms have been generated until the adoption of the actual nomenclature [6].

However, although *C. neoformans* has been recognized for more than 100 years, the worldwide spread of HIV infection as well as the increasing number of patients with impaired immunity were responsible for the dramatic surge of this life-threatening infection only in the 80's [7–9]. Although effective treatment for HIV disease has decreased significantly incidence of cryptococcal meningitis (CM) in western countries, it remains a common cause of infectious morbidity and mortality especially among HIV-positive patients living in sub-Saharan Africa and South-east Asia [10–16]. This paper will focus on main aspects of AIDS-associated cryptococcal meningitis with special attention on recent findings on immune reconstitution inflammatory syndrome, diagnosis, treatment, and prevention. Data were identified by searches on PubMed and Scopus using a combination of search terms including “cryptococcosis,” “AIDS,” “immune reconstitution inflammatory syndrome,” “cryptococcal antigen,” “cryptococcal meningitis,” “cryptococcal diagnosis,” “antifungal treatment,” “primary prophylaxis,” and “prophylaxis discontinuation.” Moreover references from relevant key articles and from my own files were included. The final search date was October 2012.

## 2. Biology, Microbiology, and Serotypes Distribution

More than 30 species of the genus *Cryptococcus* exist but only two are considered important human pathogens. The fungus is actually classified into the *Cryptococcus neoformans* complex that consists of two species: *C. neoformans* and *C. gattii* [17–19]. *C. neoformans* var. *grubii* (serotype A), *C. neoformans* var. *neoformans* (serotype D), and AD hybrids generally cause disease in immunocompromised patients whereas *C.gatti* (serotype B and C) can cause disease also in immunocompetent patients [9, 20–22].

Molecular typing has identified eight major molecular types within the 2 species: VNI/AFLP1, VNII/AFLP1A (*C. neoformans* var*. grubii*), VNIII/AFLP3 (AD hybrid), VNIV/AFLP2 (*C. neoformans* var. *neoformans*), and VGI/AFLP4, VGII/AFLP6, VGIII/AFLP5, VGIV/AFLP7 (*C. gattii*) [23].

Non-*neoformans* cryptococcal infections are rarely encountered and have been recently reviewed [24]. 

**Figure 1:**
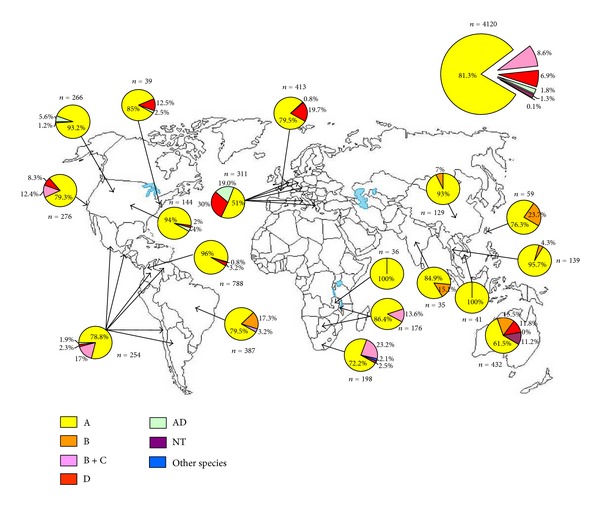
Worldwide geographic diffusion of different serotypes of *Cryptococcus neoformans* complex (based on [25–48]).

*C. neoformans* var. *grubii *has a worldwide distribution and is the most prevalent species (Figure 1) whereas *C. neoformans* var. *neoformans* is found predominantly in Western Europe [25–37]. *Cryptococcus gattii* was originally known to be geographically restricted to tropical and subtropical regions [38–40] but, since 1999, an unprecedented emergence of *C. gattii* was observed in the east coast of Vancouver Island, British Columbia where the organism is now endemic and where the incidence is the highest ever reported (37 times greater than reported in Australia) [41, 42]. Moreover, there is recent evidence that the *C. gattii* strain of the Vancouver outbreak has spread to the Pacific Northwest of the United States [43]. Finally, in recent years *C. gattii* has been isolated also in temperate regions of Europe (Austria, Italy, Spain, The Netherlands) as well as of Asia (Japan, South Korea) [44–47]. 

## 3. Epidemiology and Risk Factors

The analysis of the risk factors associated with development of cryptococcosis employed in several studies clearly demonstrates that HIV infection is the major underlying condition predisposing to the development of this disease [8, 33, 48]. Several studies have underscored the profound impact that the emergence of HIV/AIDS epidemic has had on the epidemiology of cryptococcal infection [8, 12, 13, 49, 50]; in fact, it has been reported that there were 1264 proven cases of cryptococcosis in the United States during the 12-year period from 1965 to 1977 [51]. In contrast a 1500% increase was observed in the period from 1981 to 1990 due to the AIDS epidemic; because of the introduction of azoles therapy and, by the mid-90s, of the highly active antiretroviral therapy (HAART), a declining incidence was observed in all Western countries [10–13, 52]. A completely different situation is registered in sub-Saharan Africa: worth noting an increasing numbers of cases of CM were observed among young Congolese adults starting from the early 60s possibly as the beginning of the HIV epidemic [53]. In a large part of sub-Saharan Africa as demonstrated by a recent controlled clinical trial conducted on bacterial meningitis, as well as in other studies, *C. neoformans* is now emerged as the leading cause of meningitis accounting for 26 to 45% of cases in several series [54–57]. 

It has been estimated that in 2006 at least 957,900 cases of CM had occurred worldwide with the highest burden from sub-Saharan Africa (720,000 cases with 504,000 deaths) [1] Moreover, despite the fact that also in sub-Saharan Africa, an increasing number of HIV-positive patients are now receiving HAART, a study conducted in South Africa which showed that the number of new India ink positive cases of CM was not falling [58]. However, in Burkina Faso where the incidence of HIV infection was lower and probably where better ART programs are employed, a steady decline of CM was observed during an 8-year period in parallel with the increased use of HAART [59]. Both studies are a reminder of the importance of more huge implementation of ART in Africa with a more widespread and earlier access to these life-saving therapies. 

## 4. Pathogenesis

The portal of entry of *Cryptococcus *is usually through inhalation of spores or desiccated yeast cells from the environment. Deposition in the alveoli produces an asymptomatic infection that is either cleared or controlled by a strong cell-mediated immune response leading to a dormant latent infection with development of granuloma in the lungs or hilar lymph nodes [60, 61]. In immunocompromised individuals, the fungus can spread from the lungs to the central nervous system to cause meningoencephalitis that is fatal if left untreated.Once *C. neoformans* enters the circulatory system it must survive for dissemination to occur and macrophage can be a vehicle either for dissemination and distribution through the host; crossing the blood brain barrier (BBB) is probably mediated by transcytosis (i.e., transcellular penetration through microvascular endothelial cells) and there is evidence that the capsule is involved [62, 63].

Macrophages probably play also an important role in the BBB crossing by *C. neoformans* with a mechanism of “Trojan horse”; it has been shown *in vitro* that active macrophage phagosomal extrusion is a possible escape strategy allowing continued propagation and dissemination of the fungus [64]. Recently, Charlier et al. presented proofs of concept for the hypothesis of the Trojan horse in a mouse model comparing intravenous inoculation of the yeast in a free form and after ingestion by cultured monocytes [65].

## 5. Clinical Manifestations

The fungus *Cryptococcus* can infect any organ in the body but showing a predilection for the lung and the central nervous system (CNS). The lung is the usual portal of entry of the microorganism and the infection can run either an asymptomatic course, especially among immunocompetent subjects, or progress to a life-threatening pneumonia with respiratory failure as observed in patients with AIDS [66]. 

In the pre-HAART era, cryptococcal infection was identified as the only cause of acute respiratory failure in 9% of a cohort of 210 patients with AIDS [66]; black race, lactate dehydrogenase, lactate dehydrogenase level >500 IU/L, presence of skin lesions, and interstitial infiltrates were the variables independently predictive of developing acute respiratory failure [66]. In a case series of 18 HIV-positive patients diagnosed with cryptococcal meningitis, Driver et al. found that 78% had respiratory symptoms during the 4-month period before meningitis appeared as compared with 50% at the time of diagnosis [67]. More recently,in Uganda among HIV-infected subjects hospitalized with pneumonia and undergoing bronchoscopy, the diagnosis of pulmonary cryptococcosis was made in 11% of cases [68]. Worth noting the diagnosis of pulmonary cryptococcosis was unsuspected in all these cases before diagnostic testing, thus confirming a lack of awareness of this pulmonary pathogen, as described in an autopsy study conducted on south African miners [69]. Among HIV-infected hospitalized patients with acute respiratory infections in rural Thailand, *Cryptococcus *species was found to be the second leading infection (after tuberculosis), and in 43% of all cryptococcal antigen positive, there was no evidence of past or concomitant meningitis nor any alternative etiology was identified [70].

*C. neoformans* shows a predilection to invade the central nervous system causing meningitis or meningoencephalitis in about 77–86% of patients [2, 71–89]; usually CM presents itself with a subacute or chronic course but occasionally may be fulminant [71]. Signs and symptoms of presentation may vary according to the host with fever and headache more frequently observed in the HIV-positive subjects (Table 1) and altered mental status among HIV-negative people [2, 7, 20, 27, 28, 55–57, 72–90]. In the HIV-positive patient CM is a late opportunistic infection usually observed when the T lymphocyte CD4+ cell count falls below 50–100/*μ*L; two-thirds of patients had less than 50 CD4+ cells/*μ*L and the median value reported in several series is below 30/*μ*L [2, 77, 79].

Meningismus or neck stiffness is observed globally in about 61% of cases but more frequently in HIV-positive patients in Africa [28, 55–57, 80–85] compared to HIV-positive patients in Western countries [7, 20, 71–77] or HIV-uninfected patients [2, 20, 27, 79, 87, 89, 91]. However, this finding may reflect a delayed presentation in sub-Saharan Africa in comparison with patients observed in western countries. Altered mental status, which is the most important predictor of poor outcome of patients with CM, is present in 18–28% of patients with AIDS and in half of those without HIV infection (Table 1) [72–90]. Fungemia, expression of disseminated disease is observed in 47–71% of HIV-positive patients but only in 27% of HIV-negative subjects affected by cryptococcosis. Ocular involvement with visual changes is reported in near one-third of HIV-infected patients. Several manifestations can occur such as oculomotor palsies, retinal hemorrhages, and endophthalmitis [91–94]. Vision loss may be due to yeasts infiltration of optic nerve or may be caused by increased intracranial pressure [95]. Permanent blindness was associated with visual loss within the first week of hospital admission [94]. 

**Figure 2:**
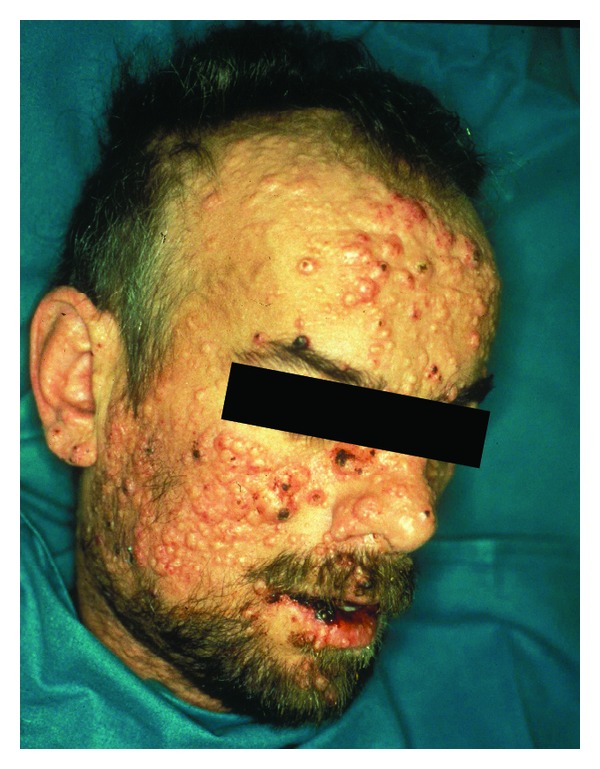
Patients with cryptococcal meningitis and disseminated infection with multiple papular and molluscum-like lesions on the face.

Skin lesions due to *C. neoformans* are found in 6% of AIDS patients with CM but the frequency is higher in patients infected with serotype D due to the seemingly higher skin tropism of this serotype [96–98]. Skin lesions are usually expression of the haematogenous dissemination of the fungus and can be single or multiple and present with a wide variety of pictures (papules, pustules, vesicles, nodules, ulcers) although a molluscum contagiosum-like appearance is the most frequently reported (Figure 2). 

## 6. Cryptococcosis in Children

Cryptococcal disease seems to occur less frequently in AIDS children than in adults with a prevalence of 0.8 to 1.4% [99–101]. However, in Thailand a 2.97% point prevalence was observed during an eight-year study among hospitalized HIV-infected patients [102]. In South Africa, in the largest study so far conducted, the incidence of cryptococcosis among HIV-positive children was 47 cases per 100.000 persons [103]. Median age at time of diagnosis was 7 years that is similar to the figures (7.8 and 9.8 years) previously reported in the literature [103–105]. In the South Africa experience, there was the demonstration of the highest incidence among children with less than 1 year of age with a second peak among children in the 5-through 10-year age group [103]. 

Older studies suggested that children with HIV infection acquired by vertical transmission may be less likely to develop *C. neoformans* infection than children infected by other routes [100, 117]. However, these observations were not confirmed in those studies conducted in Africa and may reflect the fact that the predominant risk for pediatric AIDS at the beginning of epidemic was transfusion [101, 102]. 

Clinical features in children were similar to those observed in adults but those diagnosed in Africa showed higher frequency of headache (86% versus 53%), nuchal rigidity (69% versus 7%), seizures (38% versus 13%), and focal neurologic signs (23% versus 7%) in comparison with patients observed in USA [100, 101].

## 7. Elevated Intracranial Pressure

Elevated intracranial pressure (ICP) is a common complication of AIDS-associated CM. The pathophysiology of elevated ICP in patients with CM is poorly understood and it is likely caused by several convergent factors. It has been postulated that it may be due to the outflow obstruction precipitated by aggregation of fungal polysaccharide accumulating in arachnoid villi and subarachnoid spaces thus providing the blockage of the channels for CSF drainage [118]. This picture is consistent with communicating hydrocephalus. Moreover, another possible important factor is cerebral oedema resulting from cytokine-induced inflammation and possibly osmotic effect by fungus-derived mannitol [119].

In an old observational study about 60% of the patients had an intracranial pressure of ≥250 mm H_2_O, 30% of whom showed an intracranial pressure of ≥350 mm H_2_O [119]. Similar results were subsequently confirmed in two large studies showing that 19–27% of patients with CM have a CSF pressure of ≥350 mm H_2_O [120, 121]. The highest value of CSF opening pressure is consistently associated with higher fungal burden, significantly more frequent observation of papilledema, hearing loss, meningismus and increased risk of early mortality [119, 120]. However this latter finding was not confirmed in the study of Bicanic and coworkers [121].

A recent autopsy study elegantly demonstrated a correlation between high concentration of viable and dead organisms in the arachnoid granulations and raised cerebrospinal fluid pressure with the consequence of the obstruction of CSF resorption [122].

The optimal management of elevated ICP without hydrocephalus in the setting of CM is still uncertain and debated. The updated guidelines by the Infectious Diseases Society of America on the basis of the study by Graybill and coworkers recommend, after appropriate brain imaging to exclude hydrocephalus or space-occupying lesions, CSF drainage by lumbar puncture (with a BII evidence) [123]; if symptoms persist with concomitant high CSF pressure (>250 mm H_2_O) daily drainage by repeated lumbar puncture or positioning of temporary percutaneous lumbar drains is recommended (with a BIII evidence). However, daily lumbar punctures although associated with immediate relief of headache may be associated with the risk of brain herniation [124]. 

The placement of lumbar drains has been proposed for control of raised ICP when the more conservative measures have failed; this technique allows the removal of more cerebrospinal fluid than can be safely removed by daily lumbar puncture [125, 126]. The use of temporary lumbar drainage has been used also in resource-poor setting without significant increase of bacterial infections [127]. Finally, the use of permanent ventriculoperitoneal shunts can be reserved for patients with true hydrocephalus or raised ICP refractory to all other measures employed [128].

## 8. Cryptococcal Immune Reconstitution Inflammatory Syndrome (IRIS)

Cryptococcal immune reconstitution inflammatory syndrome (IRIS) can be associated with a clinical deterioration of the disease or with a new presentation of cryptococcal disease after reversal of the immune deficiency state [129, 130]. Cryptococcal IRIS has been initially described in HIV-infected patients after starting highly active antiretroviral therapy (HAART) but it has been subsequently recognized also in the transplantation setting and following treatment with adalimumab and alemntuzumab [131–133]. Recently a group of experts on behalf of the International Network for the Study of HIV-associated IRIS (INSHI) proposed the adoption of two clinical case definitions of IRIS associated with cryptococcosis among HIV-infected patients: (1) paradoxical cryptococcal IRIS involving patients who had a diagnosis of cryptococcal disease before starting ART; (2) ART-associated cryptococcosis characterized by incident or unmasking cryptococcosis after starting antiretroviral treatment [134]. 

The incidence of paradoxical IRIS associated with cryptococcosis in AIDS patients was reported to be 15.9 events/100 patient-years of follow-up (95% CI 8.5–22.9) in one study from USA, 4.2/100 person-years(PY) of follow-up (95% CI 2.2–7.8) in another study conducted in France, and 25/100 PY in South Africa [135–137].

On the other hand, the incidence of unmasking (or ART-associated) IRIS is lower ranging from 0.2% to 1.6% as reported in two retrospective and three prospective studies [110, 137–140].

A meta-analysis of 54 cohort studies comprising more than 13.000 patients starting HAART indicated that CM was associated with the development of IRIS in 19.5% of cases (95% CrI 6.7–44.8%). It ranked second among the opportunistic infections more frequently associated with IRIS but, more notably, had the highest mortality attributed to the syndrome (20.8%) [141]. However, the study by Muller et al. was criticized because it failed to distinguish paradoxical from unmasking IRIS with the possible underestimate of the true frequency of paradoxical-cryptococcal IRIS [142]. The differences in the incidence of cryptococcal IRIS reported across different studies probably reflect the typology of the study (retrospective versus prospective), different clinical definitions employed, and different settings (resource-limited countries versus high-income countries). However, what is undoubted is the fact that cryptococcal IRIS is a true problem with an high expected burden especially in sub-Saharan Africa, Southeast Asia and Latin America [143]. The pathogenesis of cryptococcal IRIS is complex and not fully understood but is mainly attributable to the severe immunodeficiency that is unable to clear the foreign antigens and the subsequent cytokine storm induced by the restored inflammatory response [144].

Another controversial point is the establishment of the period of time during which the patients are at risk of developing cryptococcal IRIS. In fact, the time of appearance of IRIS varied quite widely among the different studies with the shortest interval after the start of HAART reported to be few days and the longest 22–27 months [145–148]. However, in four studies (two retrospective and two prospective) performed on patients affected by cryptococcosis the median time elapsing between initiation of HAART and onset of IRIS was reported to be 30 days (range 3–330 days) [135], 44 days (range 36–62 days) [149], 63 days (range 12–129 days) [150], and 8 months (range 2–37 months) [136]. It is plausible that the observed discrepancy can be explained by differences in the clinical pictures presented with less central nervous system involvement in the study reporting the longest interval [135]. This finding is in keeping with the results of a review of 25 episodes of IRIS-associated cryptococcosis showing a median time interval of 3.5 months for patients with CNS manifestations compared to 7 months for those with lymphadenitis [151].

In the case definition elaborated by the experts of INSHI, they proposed a maximum time of 12 months for the development of paradoxical IRIS after ART initiation; although acknowledging that the cut-off chosen was largely arbitrary and would exclude those occasional cases presenting after a longer interval, they believed that it might improve the specificity and its practical usefulness [134].

In a retrospective multicentre study from France, a diagnosis of cryptococcosis revealing HIV-infection (OR 4.8), presence of fungemia at baseline (OR 6.1), CD4 cell count of less than 7/*μ*L (OR 4.0) and starting HAART within 2 months of diagnosis of cryptococcosis (OR 5.5) were identified in a multivariate model as independent risk factors for development of IRIS [136]. Another retrospective cohort study from USA showed that patients who had started HAART within 30 days after the diagnosis of cryptococcosis were more likely to develop IRIS (RR 1.73, 95% CI 1.03–2.29; *P* = .031) [135]. On the contrary, Bicanic et al. in a prospective observational study conducted in South Africa on 65 antiretroviral naïve patients affected by CM were unable to find any association with fungal burden, baseline CD4 cell count, HIV viral load and time to start of HAART, and the subsequent development of IRIS [149]. They showed that a more rapid and strong immunological response measured at 6 months (220 versus 124 CD4 cells/*μ*L, *P* = 0.01) was the only variable associated with development of IRIS [149]. Finally, in Thailand the investigators found a higher baseline cryptococcal antigen as the only risk factor associated with development of IRIS [150].

Interestingly in a prospective study conducted in a cohort of ART-naïve Ugandans that aimed to identify serum biomarkers predictive of IRIS development, a 4-fold higher baseline concentrations of cryptococcal antigen was observed among patients who developed IRIS in comparison with those who did not develop IRIS [152]. In the same study higher levels of IL-4 and IL-17 and lower levels of TNF-*α*, granulocyte colony-stimulating factor (G-CSF), granulocyte-macrophage colony-stimulating factor (GM-CSF), and vascular endothelial growth factor (VEGF) were predictive of IRIS with a receiver operating characteristic (ROC) curve of 0.82. The same group showed that a paucity of inflammation in the cerebrospinal fluid at the time of CM is associated with failure to sterilize CSF at 2 weeks and subsequent risk of developing IRIS [153].

Clinical presentation of paradoxical IRIS can range from the more frequent picture of relapsing meningitis with raised intracranial pressure and negative CSF culture [13, 130, 135, 136, 138–140, 145, 149, 150, 154–158] to other less frequent central nervous system diseases (i.e., cerebral cryptococcoma, intramedullary abscess, cerebellitis with mass effect, visual and hearing loss, and radiculitis) [135, 136, 146, 147, 159–163] or alternatively may present with extraneural pictures (Table 3). Lymphadenopathy [128, 135, 136, 145, 147, 151, 158, 164], pulmonary disease, including cavitating,necrotizing and nodular lesions [130, 136, 165], subcutaneous abscess and erosive bone lesions [129, 164, 165] are the extraneural manifestations of paradoxical cryptococcal IRIS. The clinical manifestations of ART-associated cryptococcosis (comprising also unmasking disease) are essentially similar, with a predominance of meningitis [110, 129, 135, 149], lymphadenopathy, and subcutaneous abscesses [166–170].

Cryptococcal IRIS, especially when presenting with CNS manifestation, is a potentially lethal disease [136, 140, 145, 149, 151, 157] with an average fatality of about 20% [141]. Different approaches in the management of this complication have been employed including anti-inflammatory steroidal [136, 145, 155, 156, 160, 164], and nonsteroidal therapy [164, 165], thalidomide [136], anti-TNF monoclonal antibody [171], surgery [130, 136, 147, 151], and antifungal therapy (including increased dosage, change of drug or resumed antifungal therapy) [129, 130, 151, 154, 155, 157, 159].

## 9. Laboratory Diagnosis

**Figure 3:**
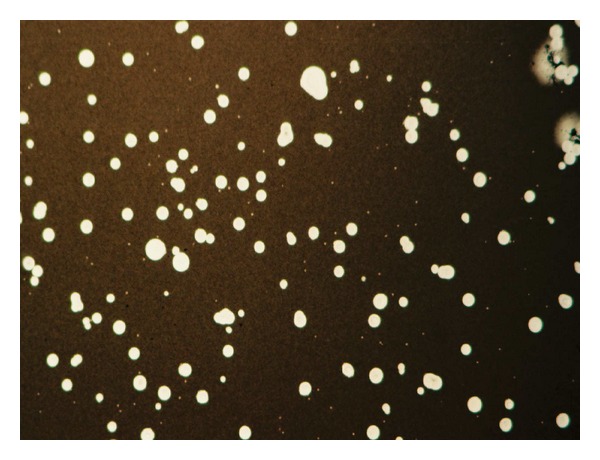
India ink preparation of cerebrospinal fluid showing yeast cells some of which budding surrounded by large capsule (Courtesy Dr Giuseppe Giuliani).

**Figure 4:**
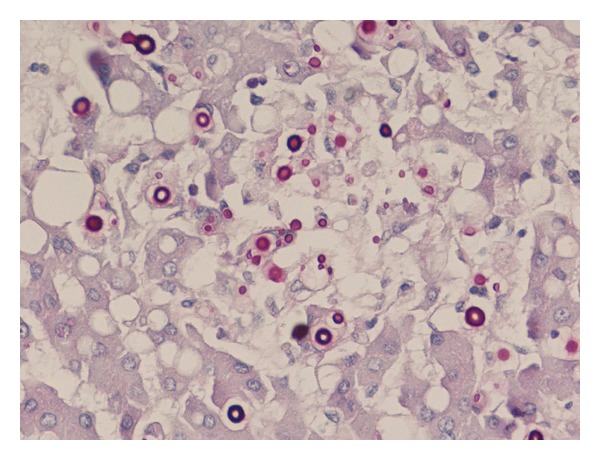
Liver histology showing PAS-positive yeast cells in a case of disseminated cryptococcosis (Courtesy Dr. Carlo Parravicini).

A definitive diagnosis of CM is made by positive CSF culture and/or direct identification of the organism by means of India ink staining (Figure 3). *Cryptococcus neoformans*, however, is able to disseminate and infect most areas of the body (i.e., lung, skin, lymph node, spleen, liver, prostate, eye, bone, bone marrow, heart, thyroid, adrenal gland, muscle) and thus it can be cultured or shown by histopathology as well from different body specimens (Figure 4). Blood cultures are reported to be positive in 47–70% of HIV-infected patients as compared to 21% of HIV-uninfected patients with CM (Table 1).

A presumptive diagnosis of CM can be made on the basis of a positive cryptococcal antigen (CrAg) using a latex agglutination or an enzyme immunoassay kit. Cryptococcal antigen testing on blood and CSF is a highly reliable and rapid diagnostic technique with excellent sensitivity and specificity [172]. Sensitivity of CSF cryptococcal antigen test is 92% to 96% in patients with culture-proven CM (Table 1). False positive results are associated with rarer infections due to *Trichosporon beigelii*, *Capnocytophaga canimorsus,* and *Stomatococcus mucilaginosus* and, on the contrary, false negative results are observed either with poorly encapsulated organisms and low fungal burden or the prozone phenomenon [173–175].

HIV-positive patients usually have very high serum and CSF CrAg titres than their HIV-negative counterpart as a reflection of the higher yeast burden and, in the pre-HAART era, both were predictive of mycological failure and poor outcome [8, 13]. In one cohort study conducted in Uganda among HIV-infected adults, detectable cryptococcal antigenemia preceded symptoms by a median of 22 days and approximately 11% of people had cryptococcal antigenaemia for more than 100 days [28].

In areas of high prevalence of cryptococcosis, the use of serum CrAg as a screening test is helpful to identify those HIV-infected people with advanced disease at risk of developing cryptococcal meningitis and who might benefit of a preemptive antifungal treatment together with antiretroviral therapy [107–110, 113]. A summary of studies reporting on detection of CrAg is reported in Table 2 [28, 106–116].

Using a cut-off value of >1 : 8 the CrAg assay was 100% sensitive and 96% specific for predicting incident CM during the first year of HAART [109]. Subsequently, in a prospective observational study conducted in Kampala (Uganda), Meya and coworkers showed that CrAg testing targeting people with severe immunodeficiency (less than 100 CD4+ cell/*μ*L) was a cost-effective measure showing 71% survival at 30-month of those treated with fluconazole together with HAART compared with 100% deaths within 2 months in those treated only with HAART [110]. 

In 2009, a new lateral flow assay (LFA) characterized by rapidity (5–15 minutes), stability at room temperature, and affordability (1.5 to 2.5 US $) was developed for the detection of cryptococcal antigen on serum, CSF, and urine [198]. All the above mentioned characteristics made it an ideal test to be used at point-of-care (POC) in resource-limited countries. This dipstick assay has been evaluated and validated against EIA positive sera and *Cryptococcus* positive blood culture from HIV-positive patients from Thailand, showing sensitive ranging from 96% to 100%, respectively [199]. In another retrospective study using serum, plasma and urine from patients with HIV-associated cryptococcal meningitis sensitivity was 100% for serum and plasma and 98% for urine [200]. It has been suggested that the ratio of titres measured by latex agglutination (LA) in comparison with LFA is 1 : 5 [201] but a small study found imperfect agreement (LFA:LA 1.53 with confidence limits from 0.13 to 18.1) [202]. Based on all the above mentioned studies, it has been proposed to integrate CrAg as a screening test into routine HIV care not only in resource-limited settings but also in western countries [200, 202, 203].

Finally two studies showed cross-reactivity between the Platelia *Aspergillus* galactomannan assay and serum from patients with cryptococcal meningitis with positive results ranging from 13.6 to 63.3% [204, 205]. 

## 10. Prognosis and Outcome

Left untreated CM is a uniformly fatal disease but even with antifungal treatment the outcome is influenced either by factors associated with the host and the underlying diseases and the fungus [206]. Several studies have confirmed that abnormal mental status [14, 74, 207], high cerebrospinal fungal burden (demonstrated by high cryptococcal antigen titers or colony forming unit) [20, 74, 76, 77, 122, 183, 184, 207], disseminated infection [20], wasting syndrome (low body mass index) [83] symptomatic elevated ICP [121], low CSF leukocyte cell counts [14, 71], lack of flucytosine treatment during induction phase [20], induction treatment with low dose fluconazole [82, 85, 207] are factors associated with poor outcome of AIDS-related CM.

In the Antiretroviral Therapy Cohort Collaboration study (including 15 cohorts with over 31,000 HIV-positive patients without prior AIDS-defining events who started HAART), cryptococcosis ranked third (after non-Hodgkin lymphoma and progressive multifocal leukoencephalopathy), as a cause of death overall and within the first 6 months after the beginning of therapy [208]. However, the introduction of HAART has witnessed a marked increase of survival of patients with cryptococcal meningitis either in resource poor- and in high-income countries [13, 14, 209]. The 14-day survival rates among antiretroviral-naïve persons with CM was 49% in 2001-02 and 80% in 2006 in Kampala, Uganda [14]. However, the 1-year survival rate in Uganda remains 4-fold worse than in France and 2-fold worse than in Thailand even with the availability of HAART [20, 210]. Another important point is the still high early mortality associated with CM that is apparently not influenced by HAART: 18% probability of death versus 21% at 3 months, respectively, in the pre-HAART and post-HAART period in France or 15% deaths at 2 weeks and 31% at 10 weeks in four cohorts in Thailand, Uganda, and South Africa [209, 210].

## 11. Antifungal Treatment

Several clinical trials or cohort studies regarding antifungal therapy for AIDS-associated CM have been performed and published over the last 22 years and are summarized in Table 4 [74, 76, 85, 177–182, 185–197]. However, it is worth noting that much of these studies have been hampered by several drawbacks such as low number of patients enrolled, low dosages of antifungal employed, different end-points, short follow-up. Current management of AIDS-associated CM is largely based on the results of the landmark randomized trial published over a decade ago [76]. It is based on three distinct phases: induction, consolidation, and maintenance therapy. The induction phase is aimed to achieve rapid fungicidal activity to obtain CSF sterility using the combination of amphotericin B deoxycholate (0.7 mg/kg/day) and 5- fluorocytosine (100 mg/kg/day) for two weeks. The combination of amphotericin B deoxycholate (AmB) and fluorocytosine (5-FC) is more rapidly fungicidal than amphotericin B alone and is associated with a trend towards a higher proportion of patients achieving a sterile CSF at 2 weeks and a reduced relapse rate [76]. The consolidation phase is the subsequent 8-week treatment period with either fluconazole (400 mg/day) or itraconazole (400 mg/day) as an alternative for patients with fluconazole intolerance. The maintenance phase or secondary prophylaxis of treatment for CM in HIV-infected subjects is imperative in order to prevent the high relapse rate of this meningitis. Oral fluconazole at a dosage of 200 mg/day has been shown to be superior to weekly intravenous AmB [211], placebo [212], or oral itraconazole [213]. Although the above mentioned treatment is endorsed by IDSA guidelines as the recommended first line treatment of AIDS-associated CM [123], it is far from being completely satisfactory because during the induction phase, it is unable to achieve CSF sterility in all patients and it is associated with a still high acute mortality; moreover, one of its component (i.e., 5-FC) is not available in most developing countries where CM is prevalent. One of the goals that needs to be achieved in the treatment of CM, especially among immunocompromised patients, is that of rapidly and efficiently reduce the huge burden of yeast cells in the CSF. In this regard in an elegant randomized study performed in Thailand, using for the first time the rate of reduction in CSF colony forming units from serial quantitative CSF cultures, Brouwer and coworkers demonstrated that the combination of AmB plus 5-FC had the highest and most rapid fungicidal activity compared to AmB alone, AmB plus fluconazole or all the three drugs together [183]. Since AmB shows concentration-dependent antifungal activity, the same group conducted a randomized controlled trial in 64 HIV-infected antiretroviral naïve patients to compare the standard of care with a higher drug dosage (i.e., 1 mg/kg/day) [185]. Although they showed that the higher dosage had a significantly greater early fungicidal activity compared to the standard dose (−0.56 log cfu/mL versus −0.45 log cfu/mL, *P* = .02), mortality at 2 and 10 weeks was not statistically different in the two arms [185].

However, analyzing combined data of early fungicidal activity collected from different cohorts, Bicanic and coworkers were able to show that the magnitude of this was inversely associated with mortality at 2 and 10 weeks and in a multivariate models (that included altered mental status and fungal burden), it remained independently associated with mortality [207]. As discussed by the Authors it is unknown if this relationship is linear or if exists a threshold value above which no further benefit of early fungicidal activity will be observed. 

In consideration of the fact that 5-FC is presently unavailable in several resource-limited areas where, on the contrary, fluconazole might be obtained free of charge through the Pfizer program or as a cheap generic drug, a phase II randomized open label trial was conducted in Thailand and USA comparing three arms (AmB at 0.7 mg/kg versus AmB at the same dosage plus fluconazole 400 mg or 800 mg per day) [188]. Using a composite efficacy end-point (CSF negative culture, stable neurological function, and survival) the arm employing higher dosage of fluconazole showed the better response (53.7 versus 28.3 versus 42.5) at 2 weeks [188]. The 2011 WHO guidelines for the management of cryptococcal disease in HIV-infected subjects strongly recommend treatment with amphotericin-based regimens [214] but in the real life a strategy employing fluconazole monotherapy is generally used. High-dose fluconazole (1200 mg/day) plus 5-FC with or without a short-course (7 days) of AmB has been shown to significantly enhance early fungicidal activity and more rapid fungal clearance form CSF although the studies were not powered for clinical end-points [192, 197].

A very interesting decision analysis study based on the results of cryptococcal trials in resource-limited areas and expected life assumptions, provided some evidence that a short-course (1-week) of AmB (1 mg/kg/d) associated with high-dose fluconazole (1200 mg/d) for 2 weeks is the most cost-effective induction treatment among the 6 regimens evaluated [215].

The French CryptoA/D prospective observational study performed on both HIV-positive or negative patients with a first episode of CM showed that also in the “real life” the best regimen for induction therapy is the combination of AmB and 5-FC [20]. Treatment failure at week 2 was documented in 26% of patients receiving AmB+5-FC in contrast with 56% with any other treatments (*P* < 0.001). Moreover, a 5-FC prescription shorter than 14 days was one of the variables independently associated with treatment failure at 3 months of follow-up confirming the results of the study of van der Horst [20, 76]. Interestingly the Authors documented that only 49% of French clinicians adhered to the recommendations of the IDSA guidelines to use the combination therapy for meningoencephalitis [20]. In a multivariate analysis the use of AmB+5-FC rather than AmB was independently associated with a high CSF CrAg titre whereas the prescription of AmB+5-FC rather than fluconazole was independently associated with high CSF CrAg titre and younger age. One might suppose that older patients are treated with fluconazole because of the better profile of tolerability but this choice exposes patients to a risk of worst outcome as it has been clearly documented.

Although AmB is still recommended as the drug of choice for induction therapy of CM by authoritative guidelines [123, 214], its use is frequently complicated by infusion-related adverse effects and nephrotoxicity that can affect mortality [216]. Results from small studies have indicated that liposomal amphotericin B (L-AMB) may represent a safer alternative to AmB with similar mycological success rates [179, 181]. A multicenter double-blind randomized comparative study performed in USA and Canada using 2 different dosages of L-AMB (3 and 6 mg/kg/day) versus conventional AmB (0.7 mg/kg/day) found similar results for primary efficacy end-point (negative CSF culture at week 2) and in the mortality rate in the 3 groups of patients (13.9%, 9.5%, and 11.5%, respectively, in the 3 and 6 mg of L-AMB and in the conventional AmB) [189]. Patients treated with L-AMB had a significantly lower incidence of infusion-related reactions compared with those treated with AmB (*P* < .001) and less nephrotoxicity with the lower dosage of L-AMB (*P* = .004). Thus the results of this study confirmed that the main advantage using L-AMB instead of AmB in the treatment of AIDS-associated cryptococcosis is a better tolerability without any significant improvement in the outcome. Recently the expert committee panel of the British HIV Association recommended the use of L-AMB (4 mg/kg/d) as preferred amphotericin B preparation for the induction therapy of AIDS-associated CM [217]. On the basis of economic considerations, other UK experts argued strongly against this endorsement [218]. Finally, two new questions, that will be discussed in the next session, emerged as the consequence of the implementation of very efficacious antiretroviral therapy in the HIV setting: (1) the appropriate time of starting ARV (antiretroviral) therapy after the diagnosis of CM; (2) the time and threshold of CD4+ lymphocytes at which maintenance therapy can be safely discontinued.

## 12. When to Start ARV Therapy and When Discontinue Antifungal Maintenance Therapy

Among HIV-infected patients with advanced disease such as those with a diagnosis of CM, there is general agreement that ARV therapy should be started as soon as possible since its deferral is associated with further HIV-related diseases and increased mortality. However, the risk of severe and potentially fatal IRIS associated with an unsterilized CSF infection is an argument against an early or concomitant (with antifungal therapy) ARV therapy. An open-label randomized strategy study of early (within 2 weeks) versus deferred (after acute therapy for opportunistic infection was completed) ARV therapy among subjects with different opportunistic infections demonstrated that the early strategy was associated with fewer AIDS progression and death [219]. However this study was underpowered with to regard CM and presented a low incidence than expected of IRIS (7%). In sharp contrast with the results of the above mentioned study, another open-label prospective study conducted in Zimbabwe showed a 3-fold greater risk of mortality in the early ARV group (within 72 hours after CM diagnosis) in comparison with the delayed ARV group (after 10 weeks of treatment with fluconazole) [220]. Worth noting the Cryptococcal Optimal ART Timing (COAT) study conducted in Uganda and South Africa under the US National Institute of Health sponsorship was ended prematurely after an interim analysis found a 1.7 times worse survival observed among patients receiving early ARV treatment (http://www.niaid.nih.gov/news/newsreleases/2012/Pages/COAT.aspx). 

It has been supposed but not proved that the fungistatic effect of fluconazole used in one study [220] as opposed to the fungicidal action of AmB used in the other study [219] could account for an increased risk of IRIS [221]. On the ground of this uncertainty guidelines offer options to start ARV therapy between 2 and 10 weeks after antifungal therapy [123, 214, 222]. The conditional recommendation of the WHO guidelines that indicates a different time option of deferred ARV initiation according to the drug regimen used in the induction and consolidation treatment seems reasonable [214].

The question regarding when antifungal maintenance therapy can be safely discontinued in patients responding to ARV therapy and at what threshold of CD4+ lymphocytes it should be done has been so far addressed in five case studies [223–227], three observational cohorts [228–230], and in one randomized controlled trial [231]. A total of 227 patients have been evaluated following discontinuation of prophylaxis: they had a median CD4+ cell count ranging from 167 to 297 cells/*μ*L, a median duration of HAART ranging from 6 to 42.5 months and a median follow-up ranging from 9 to 28.4 months after discontinuation [232]. The calculated incidence of relapse was very low and varied from 0 to 1.53 per 100 PY thus providing the basis for the indication of discontinue maintenance therapy after at least 12 months of antifungal therapy with undetectable HIVRNA (>3 months) and a sustained CD4+ cell count > 100 cells/*μ*L [123].

## 13. Prevention

In the pre-HAART era, a randomized clinical trial comparing fluconazole 200 mg/day with clotrimazole troches for prevention of fungal infections in AIDS patients demonstrated a sevenfold reduction in the risk of cryptococcosis [233]. Subsequently several other studies some of which not designed for cryptococcal prevention demonstrated that weekly administration of fluconazole at different dosages (ranging from 100 mg up to 400 mg) was successful as primary prophylaxis of CM in patients with advanced HIV disease [234–236]. However, all these studies were performed in the pre-HAART era and the policy of primary prophylaxis for CM was not recommended because of the lack of evidence of any survival benefit, the high cost associated and the risk of development of fungal resistance.

In contrast with the above mentioned lack of survival benefit a randomized double-blind placebo-controlled study performed in Thailand showed a reduction of overall mortality (HR 4.3) in patients receiving weekly fluconazole (400 mg) compared to placebo although the study had a low statistical power with wide confidence intervals for the mortality benefit (0.9–19.8) [237]. A case-control study performed in a metropolitan area of United States characterized by a high prevalence of indigent population and with a low prevalence of HAART use (12–23%) showed a protective role of azole use in preventing invasive cryptococcal disease [238].

Modelling of data from a high endemic region for cryptococcosis in resource-limited settings suggested that screening for cryptococcal antigen with preemptive treatment was more cost-effective in patients with CD4+ lymphocytes between 51–100 cells/*μ*L with primary prophylaxis better when CD4+ are below 50 cells/*μ*L [239].

Finally, a large double-blind randomized placebo controlled trial conducted in Africa provided evidence that fluconazole (200 mg) three times weekly was effective as primary prophylaxis against cryptococcal disease both before and during the early ARV therapy [240]. Moreover, although fluconazole therapy did not influence the all-cause mortality, it indeed reduced the mortality due to cryptococcosis. The results of this study make scientific strength to one of the possible strategy to be implemented in high-risk patients living in high-risk setting to protect against development of cryptococcal disease until immune reconstitution occurs.

## 14. Conclusions

Cryptococcal meningitis remains a major cause of morbidity and mortality among HIV-positive patients especially in resource-poor areas. ART is responsible of a dramatic improvement of outcome but it is associated with the possible onset of IRIS that is potentially fatal. In this regard, the optimal timing of ART beginning after the diagnosis of HIV-associated cryptococcal meningitis is unclear. Screening severe immunocompromised HIV-positive patients with the new lateral flow assay to detect CrAg is highly cost effective.

## Figures and Tables

**Table 1:** Clinical and mycological features of cryptococcal meningitis.

Signs and symptoms	HIV-positive patients, *n* = 1366*	HIV-positive patients, Africa, *n* = 4149^†^	HIV-negative patients, *n* = 675^#^	Total, *n* = 6190	HIV-positive children, *n* = 160^*∧*^
Headache	839/1014 (82.7)	2646/3324 (79.6)	292/500 (58.4)	3777/4838 (78.1)	40/60 (66.7)
Fever	812/1080 (75.2)	1914/3322 (57.6)	352/500 (70.4)	3078/4902 (62.8)	31/60 (51.7)
Cough	267/994 (26.9)	16/67 (23.9)	78/324 (24.1)	361/1385 (26.1)	NR
Dyspnea	119/872 (13.6)	NR	56/292 (19.2)	175/1164 (15.1)	7/47 (14.9)
Meningismus or neck stiffness	342/889 (38.5)	2240/3330 (67.3)	181/453 (39.9)	2763/4672 (59.1)	14/56 (25)
Visual changes	171/593 (28.8)	NR	2/16 (12.5)	173/609 (28.4)	1/40 (2.5)
Altered mental status	203/1080 (18.8)	937/3347 (27.9)	236/451 (52.3)	1378/4878 (28.2)	15/60 (25)
Seizures	28/456 (6.1)	304/3323 (9.1)	48/458 (10.5)	380/4237 (8.9)	9/60 (15)
Skin lesions	19/321 (5.9)	NR	6/32 (18.7)	25/343 (7.3)	1/60 (1.6)
AIDS-defining illness	540/1101 (49)	484/568 (85.2)	—	1024/1669 (61.3)	8/30 (27)

Mycology					

Positive CSF culture	1200/1274 (94.2)	3030/3364 (90.1)	381/464 (82.1)	4611/5102 (90.4)	97/110 (88.2)
Positive CSF antigen	489/530 (92.3)	1693/1757 (96.3)	432/447 (96.6)	2614/2734 (95.6)	38/62 (61.3)
Positive India ink	886/1132 (78.3)	3075/3360 (91.5)	269/456 (58.9)	4230/4948 (85.5)	44/50 (88)
Positive serum antigen	292/305 (95.7)	NR	121/141 (85. 8)	413/446 (92.6)	10/34 (29.4)
Positive blood cultures	645/1369 (47.1)	371/524 (70.8)	106/395 (26.8)	1122/3991 (28.1)	22/43 (51.2)

NR: not reported.

*References [7, 20, 72–80].

^†^References [28, 55–57, 81–86].

^#^References [20, 79–81, 87–91].

^*∧*^References [100–103, 105].

**Table 2:** Summary of published studies regarding cryptococcal antigenaemia in HIV-positive patients.

Author, year of publication, reference	Country	Type of study/Population	CrAg asymptomatic prevalence	All CrAg positive	Comment
Africa					

Desmet et al., 1998 [106]	Kinshasa, Zaire	Cohort/450 adults	15/450 (3.3%)	55/450 (12.2%)	29 out of 44 had CSF positive microscopy/culture for *C. neoformans *
French et al., 2002 [28]	Entebbe, Uganda	Cohort/1372 adults	7/77 (9.1%)	77/1372 (6.6%)	CrAg preceded onset of symptoms by a median of 22 days
Tassie et al., 2003 [107]	Mbarara, Uganda	Cohort/197 adults	8/197 (4.1%)	21/197 (10.7%)	13 pts had CM
Liechty et al., 2007 [108]	Kampala, Uganda	Retrospective/395 adults	22/377 (5.8)	44/395 (11.1)	7 pts newly diagnosed with CM, 3 of whom had a positive serum CrAg at baseline; asymptomatic CrAg independent risk factor for death (RR 6.6)
Jarvis et al., 2009 [109]	Cape Town, South Africa	Retrospective/707 adults	25/707 (3.5)*	46/707 (7%)	13 out of 46 (28%) developed new or relapsed CM; CrAg independent risk factor for death (HR 3.1)
Meya et al., 2010 [110]	Kampala, Uganda	Prospective cohort/609 adults	33/592 (5.6%) 26/295 (8.8% incidence) < 100 CD4+/*μ*L 7/298 (2.3%) > 100 CD4+/*μ*L	50/609 (8.2%)	17 had pre-Art diagnosed CM; preemptive treatment cost-effective (NNTac 6.5; NNTad 10)
Mamoojee et al., 2011 [111]	Kumasi, Ghana	Retrospective/92 adults	2/92 (2%)	2/92 (2%)	This study suggested a limited value of CrAg testing in this area
Oyella et al., 2012 [112]	Kampala, Uganda	Cross-sectional/367 adults	45/367 (12.2%)	69/367 (18.8%)	Only 30 pts undergo lumbar puncture; 24 out of 30 had CM

South-east Asia					

Micol et al., 2007 [113]	Phnom Penh, Cambodia	Prospective cohort/327 adults	10/327 (3.1%)	58/327 (17.7 %)	41 pts had CM
Pongsai et al., 2010 [114]	Bangkok, Thailand	Retrospective/131 adults	8/131 (6.1%)	12/131 (9.2%)	3 out of 12 had CM, 1 pulmonary cryptococcosis

Europe and USA					

Feldmesser et al., 1996 [115]	USA	Case series/10 adults	7/10 (70%)	10	First study about isolated CrAg
Patel et al., 2013 [116]	London, UK	Retrospective/157 adults	1/157 (0.6%)	7/157 (4.4%)	7 pts diagnosed with CM

CrAg: cryptococcal antigenemia, CM: cryptococcal meningitis, UK: United Kingdom; RR: relative risk; HR: hazard ratio; NNTac: number needed to treat to avoid a case; NNTad: number needed to treat to avoid death.

*Only patients without a history of cryptococcal meningitis were considered.

**Table 3:** Clinical manifestations of paradoxical and ART-associated cryptococcosis.

Clinical syndrome	References
Paradoxical cryptococcal IRIS	

Meningitis	[13, 130, 135, 136, 138–140, 145, 149, 150, 154–158]
Other CNS-IRIS manifestations	
(i) Intracranial space-occupying lesion/s	[136, 146, 159, 160]
(ii) Meningoradiculitis	[163]
(iii) Hearing loss	[161]
Lymphadenopathy	[135, 136, 145, 147, 151, 158, 164]
Cutaneous and/or soft-tissue lesions	[130, 165, 176]
Pneumonia or pulmonary nodules	[130, 136, 145]

ART-associated cryptococcal IRIS	

Unmasking meningitis	[129, 135]
Lymphadenopathy	[166, 169]
Cutaneous and/or soft-tissue lesions	[168, 170]
Pneumonia	[167]

**Table 4:** Summary of main clinical studies of antifungal induction phase for AIDS-associated cryptococcal meningitis since 1990.

Author, year of publication, reference	Study design/location	Drugs	Duration of treatment	Number of cases versus comparators	Clinical response (%)	Mycological response (%) or (EFA)^*∧*^	Overall (clinical + mycological) response (%)	Mortality (%)
Larsen et al., 1990 [177]	RCT/USA	Flu (400 mg) versus AmB (0.7 mg/kg) for 1 week then three-times weekly + 5FC (150 mg/kg)	10 weeks	14 versus 6	63	75	43 versus 100	19 versus 0 (*P* = 0.27)

Saag et al., 1992 [74]	RCT (2 : 1)/USA	Flu (200 mg) versus AmB (0.4 mg/kg)	10 weeks	131 versus 63	60 versus 67	43 versus 46 (10 weeks)	44 versus 40	15 versus 8 (2 weeks) 14 versus 18

De Gans et al., 1992 [178]	RCT (non-blinded)/The Netherlands	Itra (400 mg) versus AmB (0.3 mg/kg) + 5FC (150 mg/kg)	6 weeks	12 versus 10	NR	NR	42 versus 100	0

Coker et al., 1993 [179]	OS/Multicentre (Europe)	LAMB (4 mg/kg/d)	2 weeks	23	74	66.7	NR	0

Menichetti et al., 1996 [180]	Cohort/Italy	Flu (800–1000 mg/d)	3 weeks	14	54.5 (10 weeks)	NR	67.1	18.2

van Der Horst et al., 1997 [76]	RCT/USA	AmB (0.7 mg/kg) + 5FC (100 mg/kg) versus AmB (0.7 mg/kg)	2 weeks	202 versus 179	78 versus 83	60 versus 51 (2 weeks)	NR	Overall 5.5 (2 weeks); 3.9 (10 weeks)

Leenders et al., 1997 [181]	RCT/The Netherlands	L-AmB (4 mg/kg) versus Amb (0.7 mg/kg)	3 weeks	15 versus 13	87 versus 83 (10 weeks)	73 versus 38 (3 weeks)		Overall 10.7

Mayanja-Kizza et al., 1998 [182]	RCT/Uganda	Flu (200 mg/d) + 5FC (150 mg/kg/d) versus Flu (200 mg/d)	8 weeks	30 versus 28	NR	NR	32 versus 12	60 versus 84 (2 weeks); 68 versus 88 (6 months)

Brouwer et al., 2004 [183]	RCT/Thailand	AmB (0.7 mg/kg) + FC (100 mg/kg) versus AmB (0.7 mg/kg) versus AmB (0.7 mg/kg) + Flu (400 mg) + FC (100 mg/kg) versus AmB (0.7 mg/kg) + Flu (400 mg)	2 weeks	15 versus 16 versus 16 versus 16	NR	−0.54 versus –0.31 versus –0.38 versus –0.39^*∧*^	64 (10 weeks)	Overall 14.6 (2 weeks); 21.9 (10 weeks)

Schaars et al., 2006 [85]	Retrospective chart review/South Africa	Flu (200 mg/d) year 1991–2000 Flu (400 mg/d) 2001		77 versus 128	NR	NR	NR	23.4 versus 26.6

Bicanic et al., 2007 [184]	POT/South Africa	AmB (1 mg/kg) versus Flu 400 mg	10 weeks	49 versus 5	67 versus 25*	−0.48 versus –0.02^*∧*^	38 versus 0^†^	Overall mortality 17 (2 weeks); 37 (10 weeks)

Bicanic et al., 2008 [185]	RCT/South Africa	AmB (1 mg/kg) + 5FC (100 mg) versus AmB (0.7 mg/kg) + 5FC (100 mg)	2 weeks	34 versus 30	74 versus 79*	−0.56 versus –0.45^*∧*^	60^†^	3 versus 9 (2 weeks); 21 versus 26 (10 weeks). Overall mortality 24 (10 weeks) and no differences between groups

Longley et al., 2008 [186]	Cohort/Uganda	Flu (800 mg) versus Flu (1200 mg) then 400 mg	2 weeks + 8 weeks	30 versus 30	NR	−0.07 versus –0.18^*∧*^	40 versus 52*	37 versus 22 (2 weeks) 60 versus 48 (10 weeks)

Dammert et al., 2008 [187]	Cohort/Perù	AmB (0.7 mg/kg/d)	2-3 weeks	47	NR	25 (2 weeks) 68 (10 weeks)	70 (10 weeks)	13 (2 weeks) 22 (10 weeks)

Pappas et al., 2009 [188]	RCT/USA and Thailand	AmB (0.7 mg/kg) versus AmB (0.7 mg/kg) + Flu (400 mg) versus AmB (0.7 mg/kg) + Flu (800 mg)	2 weeks	46 versus 48 versus 41	42.5 versus 28.3 versus 52.3	NR	71.4 versus 84.2 versus 83.3 (10 weeks)	22 versus 17 versus 18.4 (10 weeks)

Hamill et al., 2010 [189]	RCT/USA and Canada	LamB (3 mg/kg/d) versus LamB (6 mg/kg/d) versus AmB (0.7 mg/kg/d)	2 weeks (11–21 days)	86 versus 94 versus 87	65.8 versus 75.3 versus 65.8 (2 weeks) 70.5 versus 72.9 versus 81.5 (10 weeks)	58.3 versus 48 versus 47.5 (2 weeks) 60 versus 70.7 versus 78.7	67.5 versus 73.7 versus 75.5 (10 weeks)	14 versus 9.6 versus 11.5 (10 weeks) Overall mortality at 10 weeks 11.6%

Lightowler et al., 2010 [190]	Cohort/South Africa	AmB (0.7 mg/kg/d) versus Flu (400 mg/d)	2 weeks	148 versus 28	NR	NR	Fluconazole associated with 5.1 RR of death at 28 days	Overall 28 (2 weeks); 32.3 (10 weeks)

Falci et al., 2010 [191]	Pilot/Brazil	AmB (0.7 mg/kg/d) continuous infusion (24-h) + FC (100 mg/k/d)	2 weeks	12	NR	−0.37^*∧*^	80	10 (2 weeks); 10 (10 weeks)

Nussbaum et al., 2010 [192]	RCT/Malawi	Flu (1200 mg/d) versus Flu (1200 mg/d) + FC (100 mg/kg/d)	2 weeks	20 versus 21	NR	−0.11 versus −0.28^*∧*^	NR	10 versus 37 (2 weeks); 43 versus 58 (10 weeks) Overall 48.8

Jadhav et al., 2010 [193]	RCT/India	LamB (1 mg/kg) versus LamB (3 mg/kg)	3 weeks	11 versus 15	NR	NR	36.6 versus 54	Overall 27 (10 weeks)

Loyse et al., 2012 [194]	RCT/South Africa	AmB (0.7–1 mg/kg) + 5FC (100 mg/d) versus AmB (0.7–1 mg/kg/d) + Flu (800 mg/d) versus AmB (0.7–1 mg/kg) + Flu (1200 mg/d) versus AmB (0.7–1 mg/kg) + Vor (600 mg/d)	2 weeks	21 versus 22 versus 24 versus 14	NR	−0.41 versus −0.38 versus −0.41 versus −0.44^*∧*^	NR	Overall 12 (2 weeks); 29 (10 weeks) (no statistically significant differences among groups)

Muzoora et al., 2012 [195]	Cohort/Uganda	Flu (1200 mg/d) + AmB (1 mg/kg/d)	2 weeks	30	NR	−0.31 (2 weeks)^*∧*^	NR	23 (2 weeks); 28 (10 weeks)

Jarvis et al., 2012 [196]	RCT/South Africa	AmB (1 mg/kg/d) + 5FC (100 mg/kg/d) versus AmB (1 mg/kg/d) + 5FC (100 mg/kg/d) + IFN*γ* (100 *μ*g) 2 doses versus AmB (1 mg/kg/d) + 5FC (100 mg/kg/d) + IFN*γ* (100 *μ*g) 6 doses	2 weeks	31 versus 29 versus 30	NR	−0.49 versus 0.64 versus −0.64^*∧*^	NR	32 versus 33 versus 27 (10 weeks) Overall 14 (2 weeks) 27 (10 weeks)

Jackson et al., 2012 [197]	RCT/Malawi	Flu (1200 mg/d) + AmB (1 mg/kg/d) versus Flu (1200 mg/d) + 5FC (100 mg/kg/d) + AmB (1 mg/kg/d for 7 days)	2 weeks	20 versus 20	NR	−0.38 versus −0.50^*∧*^	NR	No statistically significant differences at 2 and 10 weeks between the arms

RCT: randomized controlled trial; OS: open label study; NR: not reported; EFA: early fungicidal activity ^*∧*^expressed as mean log CFU/mL; Flu: fluconazole; amB: amphotericin B deox; Itra: itraconazole; 5FC: 5-fluorocytosine; LAMB: liposomal amphotericin B; IFN*γ*: interferon-gamma; Vor: voriconazole; *percentage of patients surviving at weeks 10: ^†^proportion of patients surviving after 1 year of followup.
